# Metal Organic Frameworks: Synthesis and Application

**DOI:** 10.3390/molecules25040960

**Published:** 2020-02-20

**Authors:** Victoria F. Samanidou, Eleni A. Deliyanni

**Affiliations:** 1 Laboratory of Analytical Chemistry, Department of Chemistry, Aristotle University of Thessaloniki; GR-54124 Thessaloniki, Greece; 2 Laboratory of Chemical and Environmental Technology, Department of Chemistry, Aristotle University of Thessaloniki, GR-54124 Thessaloniki, Greece

The concept of metal–organic frameworks (MOFs) was first introduced in 1990; nowadays they are among the most promising novel materials. MOFs belong to a new class of crystalline materials that consist of coordination bonds between metal clusters (e.g., metal-carboxylate clusters and metal-azolate clusters), metal atoms, or rod-shaped clusters and multidentate organic linkers that contain oxygen or nitrogen donors (like carboxylates, azoles, nitriles, etc.); thus, a three-dimensional structure is formed [1].

The properties of both metal ions and linkers determine the physical, structural, and morphological features of MOF networks (e.g., porosity, pore size, and pore surface). Additionally, the aforementioned as well as the chemical features of the prepared frameworks can be controlled by the solvent system, pH, metal-ligand ratio, and temperature [1].

Although MOFs were initially used in catalysis, gas storage and separation, membranes, or electrochemical sensors, they were later introduced as SPE (Solid Phase Extraction) sorbents. Initially they were applied for PAHs (Polycyclic Aromatic Hydrocarbons) in environmental water samples, but subsequently, the range of applications was expanded to the field of analytical chemistry, both in chromatographic separation and sample preparation, with great success in, e.g., SPE and SPME (Solid Phase Micro-extraction). Since then, the number of analytical applications implementing MOFs as sorbents in sample preparation approaches has increased. Τhis is reinforced by the fact that, at least theoretically, an infinite number of structures can be designed and synthesized, thus making tuneability one of the most unique characteristics of MOF materials. Moreover, they have been designed in various shapes, such as columns, fibers, and films, so that they can meet more analytical challenges with improved analytical features. Going a step further, the design and synthesis of advantageous composites or the controllable incorporation of defects has been shown to be a promising strategy with a positive impact on the desirable features and on stability/reusability [1].

The exceptional properties of MOFs have attracted the interest of analytical chemists who have taken advantage of their unique structures and features, and have already introduced them in several sample pretreatment techniques, such as solid phase extraction, dispersive SPE, magnetic solid phase extraction, solid phase microextraction, stir bar adsorptive extraction, etc. [1].

This Special Issue aims to present the recent developments in the synthesis and applications of MOFs.

The outcome is very impressive; ten manuscripts illustrate the impact of MOFs as useful tools in various fields like analytic methods, biofuels desulfurization, CO_2_ capture, and more. Research groups located in Greece, United Stated of America, Austria, Spain, Poland, Iran, India, and China have contributed one communication report, four original research articles, and five comprehensive reviews [1,2,3,4,5,6,7,8,9,10].

Yazdanparast et al. present an unusual, noncatenated, large pore, pillared paddle-wheel MOF, providing an additional datapoint to support current postulation on the factors that may influence catenation in these frameworks. This information will be useful to MOF chemists who are interested in the well-defined multifunctionality of these materials.

In their research article, Andriotou et al. report on luminescence color tuning in a lanthanide metal-organic framework (LnMOF) ([La(bpdc)Cl(DMF)] (**1**); bpdc^2−^ = [1,1′-biphenyl]-4,4′-dicarboxylate, DMF = *N*,*N*-dimethylformamide) by introducing dual emission properties in a La^3+^ MOF scaffold through doping with the blue fluorescent 2,2′-diamino-[1,1′-biphenyl]-4,4′-dicarboxylate (dabpdc^2−^) and the red emissive Eu^3+^.

Giannakoudakis. and Bandosz in their research article, describe the building of MOF nanocomposites with oxidized graphitic carbon nitride nanospheres. A composite of the two most studied MOFs, i.e., copper-based Cu-BTC (HKUST-1) and zirconium-based Zr-BDC (UiO-66), with oxidized graphitic carbon nitride nanospheres was designed, synthesized, and characterized. The role of oxidized g-C_3_N_4_ during the synthesis of the composite was found to be different, depending on the geometry of the framework. In the case of the UiO-66-based composite, spherical particles were obtained after the growth of the framework around the oxidized and spherical g-C_3_N_4_ nanoparticles. For the HKUST-1-based composite, the growth of the octahedral framework units experienced geometrical constraints, resulting in more defects and the creation of mesoporosity. The formation of the composite upon the incorporation of the nanospheres led to differences in the amounts of the adsorbed CO_2_.

Liang. et al. describe the facile preparation of a metal-organic framework (MIL-125)/chitosan beads for the adsorption of Pb(II) from aqueous solutions. In their research work, a novel composite of a titanium-based, metal-organic framework (MOF) with chitosan beads was synthesized following a template-free solvothermal approach under ambient conditions; the resulting composite presented a higher remediation capability compared to pure MOF.

González-Rodríguez et al. propose the mixed functionalization of organic ligands in UiO-66. Their study is intended to prepare and characterize UiO-66 derivatives incorporating different contents of nonfunctionalized and functionalized-organic ligands, including -NH_2_ and -NO_2_ groups, in the MOF structure through the mixed-linker approach. As a second goal, the paper evaluates the influence of such modifications on the resulting material when used as a sorbent in a D-µSPE method for different target analytes in water. The selected analytes presented a low to high size (to evaluate their influence when entering or not entering the pores of the MOF), while incorporating or not incorporating polar groups in their structures (to evaluate possible interactions between MOF pore functionalities and analyte groups).

Vardali et al. illustrated some novel approaches utilizing metal-organic framework composites for the extraction of organic compounds and metal traces from fish and seafood. The authors discuss the applications of MOFs and their composites/hybrids as potential media for the extraction, detection, or sensing of organic and inorganic pollutants from fish samples, prior to their determination using an instrumental technique. Emphasis is given to the extraction of antibiotics as well as metals from fish tissue, since they are considered significant contaminants in the marine environment.

In their review, Giliopoulos et al. examine the various types of polymer/MOF nanocomposites used in biomedical applications, and more specifically in drug delivery and imaging. They focus on the different approaches followed to produce the composites, and discuss their findings regarding the behavior of the composites in each application.

Manousi et al. provide a comprehensive review of the extraction of metal ions with MOFs. The authors discuss the applications of MOFs as potential sorbents for the extraction of metal ions prior to their determination from environmental, biological, and food samples. The application of subfamilies of MOFs, such as zeolitic imidazole frameworks (ZIFs) or covalent organic frameworks (COFs), is also discussed.

Kampouraki et al., describe the use of MOFs as desulfurization adsorbents of DBT and 4,6-DMDBT from fuels. In their review, applications of MOFs and their functionalized composites for adsorptive desulfurization of fuels are presented and discussed, as well as the main desulfurization mechanisms reported for the removal of thiophenic compounds by various frameworks. Prospective methods regarding the further improvement of the desulfurization capabilities of MOFs are also suggested.

Last but not least, Manousi et al. present applications of MOFs in food sample preparation. The authors identify applications of MOFs reported in the literature, including the use of metal-organic compounds and their derived carbons as absorbents in combination with dispersive sample preparation techniques, magnetic sample preparation techniques, in-tube sample preparation techniques, and online sample preparation techniques for the analysis of complex food samples, such as milk, tea and beverages, fruits and vegetables, meat, chicken, fish, etc. [1].

This special issue is accessible through the following link:

https://www.mdpi.com/journal/molecules/special_issues/MOFs

As guest editors for this Special Issue, we would like to thank all the authors and coauthors for their contributions, and all the reviewers for their time and effort in carefully evaluating the manuscripts, making recommendations that significantly improved the quality of original submissions. Last but not least, we would like to acknowledge the editorial office of the *Molecules* journal for their kind assistance in all stages of preparing this Special Issue.

We hope that readers will find the information provided in this Special Issue interesting and helpful.
